# Cognitive phenotypes in late-onset epilepsy: results from the atherosclerosis risk in communities study

**DOI:** 10.3389/fneur.2023.1230368

**Published:** 2023-08-24

**Authors:** Anny Reyes, Andrea L. C. Schneider, Anna M. Kucharska-Newton, Rebecca F. Gottesman, Emily L. Johnson, Carrie R. McDonald

**Affiliations:** ^1^Department of Radiation Medicine & Applied Sciences, University of California, San Diego, La Jolla, CA, United States; ^2^Department of Neurology, Perelman School of Medicine, University of Pennsylvania, Philadelphia, PA, United States; ^3^Department of Biostatistics, Epidemiology, and Informatics, Perelman School of Medicine, University of Pennsylvania, Philadelphia, PA, United States; ^4^Department of Epidemiology, University of North Carolina at Chapel Hill, Chapel Hill, NC, United States; ^5^National Institute of Neurological Disorders and Stroke Intramural Research Program, Bethesda, MD, United States; ^6^Department of Neurology, Johns Hopkins University School of Medicine, Baltimore, MD, United States; ^7^Department of Psychiatry, University of California, San Diego, La Jolla, CA, United States

**Keywords:** epilepsy, phenotypes, cognition, aging, dementia

## Abstract

**Introduction:**

Cognitive phenotyping is a widely used approach to characterize the heterogeneity of deficits in patients with a range of neurological disorders but has only recently been applied to patients with epilepsy. In this study, we identify cognitive phenotypes in older adults with late-onset epilepsy (LOE) and examine their demographic, clinical, and vascular profiles. Further, we examine whether specific phenotypes pose an increased risk for progressive cognitive decline.

**Methods:**

Participants were part of the Atherosclerosis Risk in Communities Study (ARIC), a prospective longitudinal community-based cohort study of 15,792 individuals initially enrolled in 1987–1989. LOE was identified from linked Centers for Medicare and Medicaid Services claims data. Ninety-one participants with LOE completed comprehensive testing either prior to or after seizure onset as part of a larger cohort in the ARIC Neurocognitive Study in either 2011–2013 or 2016–2017 (follow-up mean = 4.9 years). Cognitive phenotypes in individuals with LOE were derived by calculating test-level impairments for each participant (i.e., ≤1 SD below cognitively normal participants on measures of language, memory, and executive function/processing speed); and then assigning participants to phenotypes if they were impaired on at least two tests within a domain. The total number of impaired domains was used to determine the cognitive phenotypes (i.e., Minimal/No Impairment, Single Domain, or Multidomain).

**Results:**

At our baseline (Visit 5), 36.3% met criteria for Minimal/No Impairment, 35% for Single Domain Impairment (with executive functioning/ processing speed impaired in 53.6%), and 28.7% for Multidomain Impairment. The Minimal/No Impairment group had higher education and occupational complexity. There were no differences in clinical or vascular risk factors across phenotypes. Of those participants with longitudinal data (Visit 6; *n* = 24), 62.5% declined (i.e., progressed to a more impaired phenotype) and 37.5% remained stable. Those who remained stable were more highly educated compared to those that declined.

**Discussion:**

Our results demonstrate the presence of identifiable cognitive phenotypes in older adults with LOE. These results also highlight the high prevalence of cognitive impairments across domains, with deficits in executive function/processing speed the most common isolated impairment. We also demonstrate that higher education was associated with a Minimal/No Impairment phenotype and lower risk for cognitive decline over time.

## 1. Introduction

Older adults represent the fastest growing population of patients with epilepsy (1–4), including those with early-onset, chronic epilepsy, and those with late-onset epilepsy (LOE) (3). The incidence of epilepsy among adults 65 years and older is approximately 1 per 1,000/year, with rates increasing as a function of age (1). As the population age continues to increase, the number of older adults with LOE is also expected to rise, thus increasing the overall global burden of epilepsy.

There is great heterogeneity in the cognitive impairments observed in individuals with LOE, which may reflect heterogeneity in etiologies. Stroke is the most common cause of LOE, followed by brain tumor, head injury, and neurodegenerative disorders (2–7). However, in approximately 13%–40% of cases the cause remains unknown. In individuals with epilepsy of unknown etiology, occult cerebrovascular disease has been proposed as an etiology given the high prevalence of vascular risk factors in this population such as hypertension and diabetes (8, 9). Another potential etiology is the shared neuropathology with neurodegenerative disease, including a bidirectional relationship between epilepsy and dementia (3, 4, 10). Specifically, several prospective and retrospective studies have reported an increased risk of dementia in individuals with epilepsy (11–16) and increased risk of epilepsy in patients with Alzheimer’s disease (AD) (17–20). Further, there is evidence of AD-related pathology in patients with epilepsy including accumulation of β-amyloid (Aβ) (3, 4, 21–23) and tau (3, 4, 23), and the APOE4 genotype has been liked to an increased risk of developing epilepsy (24, 25). Together, these diverse etiologies may be expected to manifest in different cognitive profiles and differential risk for cognitive progression.

Despite increased awareness of the elevated risk of dementia in individuals with LOE and identification of risk factors for the development of LOE, the nature of cognitive deficits in this clinical population has not been fully characterized. Although several studies have examined cognitive impairments in older adults with epilepsy (25–34), only a few studies have exclusively focused on LOE (22–24, 35–39), and most of these studies have used neuropsychological screening tools with limited sensitivity that do not enable a comprehensive analysis of cognitive profiles in this growing population.

In this study, we implement an approach called *cognitive phenotyping* to better characterize the cognitive complications observed in LOE. Cognitive phenotyping has been successfully implemented across a range of disorders including chronic epilepsy (40), mild cognitive impairment (MCI) (41, 42), multiple sclerosis (43, 44), Parkinson’s disease (45), autism spectrum disorders (46), and COVID-19 (47, 48) to better define the cognitive heterogeneity inherent in a disease. This approach is a patient-centered method that considers the pattern of scores within a comprehensive battery of tests rather than individual test scores. Individuals are aggregated into distinct groups or phenotypes based on this pattern and the relationship between disease related features (e.g., clinical characteristics, brain pathology, patient outcomes) can then be examined within and across phenotypes.

Our group has shown that in young-to-middle aged adults, the phenotyping approach better captures the heterogeneity inherent both within and across epilepsy syndromes compared to analyzing individual scores in isolation (40, 49–55). We have demonstrated that cognitive phenotypes are stable and robust across cohorts and are associated with distinct patterns of brain imaging abnormalities (49, 51, 56, 57). Furthermore, other studies have utilized the phenotype approach to examine cognitive progression (56) and postoperative cognitive decline (58). Thus, identifying cognitive phenotypes in LOE could help identify individuals at increased risk for cognitive progression and development of dementia, as well as delineate LOE subtypes that may be associated with distinct clinical, vascular, and lifestyle profiles.

In this study, we identify cognitive phenotypes in a group of older adults with LOE. We also examine the demographic, clinical, and vascular profiles across cognitive phenotypes and examine whether specific phenotypes confer increased risk for cognitive decline.

## 2. Materials and methods

### 2.1. Participants

**Figure 1:**
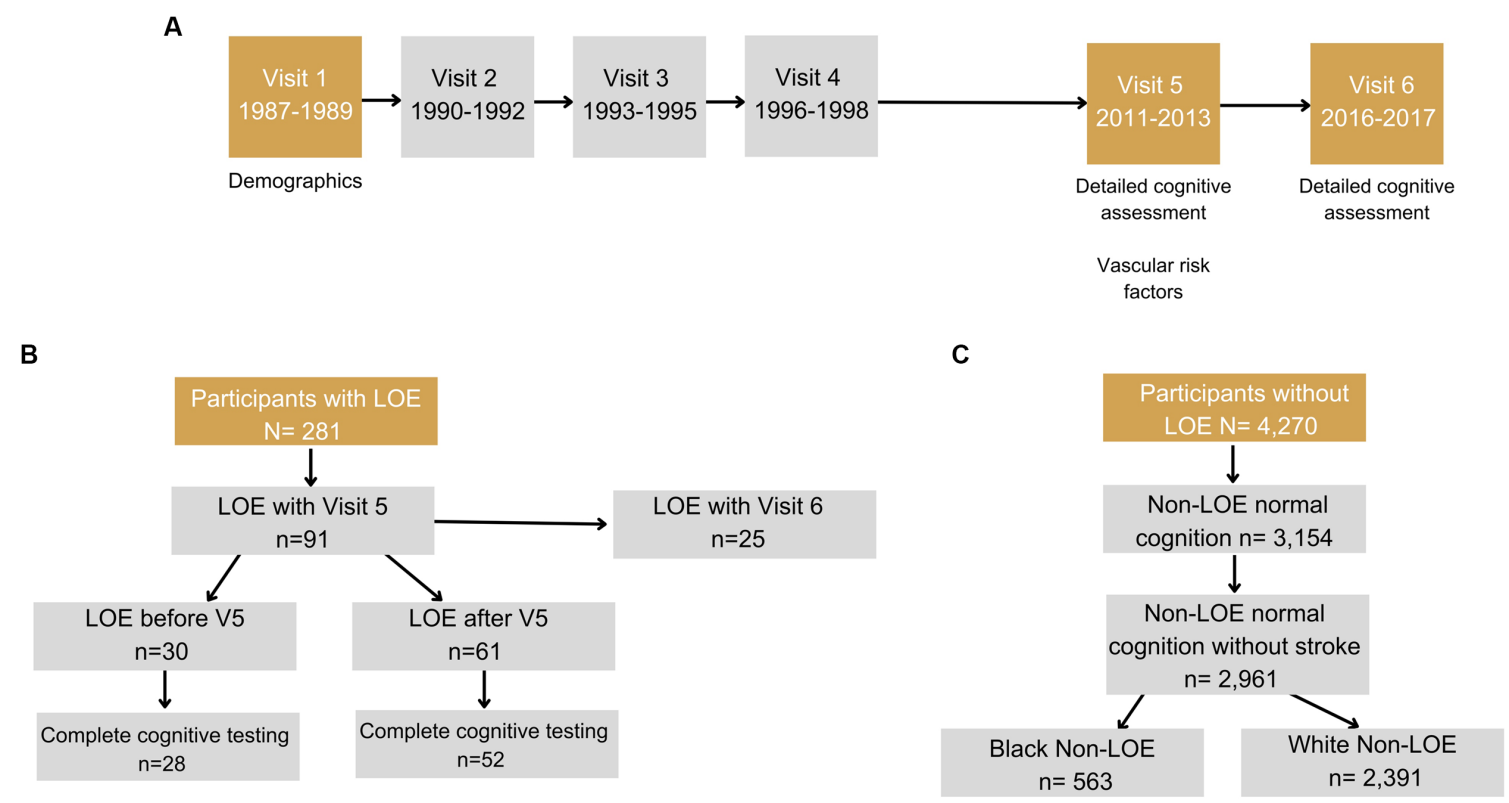
Flowcharts shows **(A)** Timeline of visits in ARIC. **(B)** Inclusion/exclusion criteria for participants with late-onset epilepsy (LOE). **(C)** Inclusion/exclusion criteria for participants without LOE and with normal cognition. Visits 2 and 4 include a short battery of cognitive tests, however, these visits were not included in the phenotyping classification.

The Atherosclerosis Risk in Communities (ARIC) study is a community-based, longitudinal cohort study of 15,792 men and women recruited from 1987 to 1989 via probability sampling from four US communities (Jackson, MS; Forsyth County, North Carolina; Washington County, Maryland; and suburbs of Minneapolis, MN) (59). Participants have completed 9 in-person visits (1987–2022) as of the time of manuscript preparation and are continuing to be followed via in-person visits and semi-annual telephone calls. For the purposes of this study, data from Visits 1 and 5–6 are included in the analyses (Figure 1). We included Black participants recruited in Mississippi and North Carolina and White participants recruited in Maryland, Minnesota, and North Carolina, and excluded participants of other races due to small sample sizes as is standard in ARIC.

### 2.2. Identification of LOE

Cases of LOE were identified in ARIC using an ICD code screening method that has been developed and validated in epilepsy (60) and previously used in ARIC (8, 16, 24, 61, 62). LOE was defined as two or more seizure-related ICD-9 or ICD-10 primary diagnostic codes (345.00–345.91: epilepsy; 780.39: seizure/convulsion; G40.0-G40.919: epilepsy; or R56.9: seizure/convulsion) identified from Centers for Medicare & Medicaid Services (CMS) fee-for-service (FFS) outpatient, inpatient, and Carrier claims from 1991 to 2018. To identify incident LOE, we included participants with at least 2 years of continuous CMS data prior to the first seizure-related code. Due to age at first CMS eligibility for most participants, the first seizure-related code had to occur at age 67 or older.

### 2.3. Demographic, clinical, and vascular risk factors

Demographic variables (i.e., race, sex, education, occupational complexity) were obtained at Visit 1. Occupation was categorized into high (managerial and professional specialty, technical, sales, and administrative support) or low (service, precision production, repair, operators, fabricators, laborers, homemakers) occupational complexities. APOE genotype was ascertained, and participants were classified as having 0, 1, or 2 Apo ε4 alleles (TaqMan assay; Applied Biosystems, Foster City, CA). Age at Visit 5 was used in the analyses. The following vascular risk factors were also ascertained at Visit 5: hypertension, diabetes, hyperlipidemia, body mass index (BMI), and alcohol use and smoking. Hypertension was defined as systolic blood pressure mean ≥ 140 mmHg (mean of second and third measurement), diastolic blood pressure mean ≥ 90 mmHg (mean of second and third measurement), or use of an antihypertensive medication. Diabetes was defined as fasting blood glucose ≥126 mg/dL, non-fasting blood glucose ≥200 mg/dL, use of diabetic medications or insulin, HbA1c > 6.5%, or self-report of physician-diagnosed diabetes. Hyperlipidemia was defined as total cholesterol ≥200 mg/dL. BMI was calculated as weight in kilograms divided by height in meters squared. Obesity defined as a BMI ≥30 was considered a vascular risk factor. Participants self-reported smoking and alcohol use (never, former, current). A burden of vascular risk score was calculated and defined by the number of vascular risk factors present (0, 1, or 2+) which included hypertension, diabetes, hyperlipidemia, obesity, and self-reported smoking. ARIC collected prevalent stroke data at Visit 1 and performs active death and hospital discharge surveillance of all cerebrovascular disease, which is adjudicated via computer algorithm and expert review (63).

### 2.4. Neuropsychological measures

All participants with LOE completed comprehensive neuropsychological testing as part of the ARIC Neurocognitive Study (ARIC-NCS) at Visit 5 and a subset of these participants completed the same battery of tests at a follow-up visit (Visit 6). Although previous ARIC studies have included a three-domain structure that includes Memory, Language and Verbal Fluency, and Sustained Attention and Processing Speed (64), we selected language, learning and memory and processing speed and executive function based on our previous phenotype study in older adults with epilepsy (33). For the purpose of the phenotype approach, processing speed and executive function were combined into one domain. Supplementary Table S1 provides full description of all the tests. Verbal memory was evaluated with the Wechsler Memory Scale-Revised Logical Memory (LM) immediate (LM1) and delayed recall (LM2) (65) and with the delayed word recall test (DWRT) (66, 67). Language ability was evaluated with the Boston Naming Test (BNT) (68), word fluency test (WFT), and animal fluency. Processing speed was assessed with the Trail Making Test condition A (TMT-A) and digit symbol substitution test (DSST) from the Wechsler Adult Intelligence Scale-Revised (69) and mental flexibility/set-shifting was measured with the Trail Making Test B (TMT-B).

### 2.5. ARIC cognitive diagnostic criteria

Mild Cognitive impairment (MCI) and dementia diagnoses in ARIC were based on the following criteria described in Knopman et al. (70); MCI was defined as at least one domain score worse than −1.5 Z, a Clinical Dementia Rating (CDR) sum of boxes >0.5 and ≤3, a Functional Ability Questionnaire (FAQ) of 5, and decline below the 10 percentile on one test or below the 20th percentile on two tests in the serial ARIC-NCS cognitive battery. Dementia was defined as >1 cognitive domain worse than −1.5 Z, a CDR sum of boxes >3 and FAQ >5, and decline below the 10 percentile on one test or below the 20th percentile on two tests in the serial ARIC-NCS cognitive battery. As described in Knopman et al. (70), cognitive normality required that all ARIC-NCS cognitive domain scores were better than −1.5 Z and that there was an absence of decline below the 10th percentile on one test or below the 20th percentile on two tests in the serial ARIC cognitive battery; and the CDR sum of boxes was required to be ≤ 0.5 and the FAQ ≤ 5.

### 2.6. *Z*-score calculation

Raw scores for all LOE participants were converted into z-scores based on data from a normal control sample stratified by race (i.e., Black and White) and education (i.e., ≤ high school, college, graduate school). The normal control sample for this study consisted of ARIC participants that did not meet criteria for LOE, had no history of stroke, and had normal cognition based on the ARIC cognitive normality definition described above. For measures with significant Shapiro–Wilk test (i.e., *p* < 0.05), extreme outliers defined as observations that fell below Q1–1.5 interquartile range (IQR) or above Q3 + 1.5 IQR were removed from the Non-LOE normal participants. Given the differences in cognitive performance (Supplementary Table S2) between the White and Black Non-LOE participants, z-scores were calculated separately for each racial group.

### 2.7. Base rates of impairment

Rates of impairment at the individual test level were calculated to examine the cognitive processes/tests that were most affected in the ARIC LOE sample. *Z*-scores from Visit 5 were classified as impaired or not impaired using a ≤ −1.0 standard deviation (SD) cutoff. The −1.0 SD was used as the test-level impairment cut-off because this cut-off has been demonstrated to balance sensitivity and stability of impairment when examining profiles of scores (i.e., phenotypes) rather than scores in isolation (71). Base rates were calculated by dividing the number of LOE participants classified as impaired on an individual test to the total number of LOE participants.

### 2.8. Identifying cognitive phenotypes

**Figure 2:**
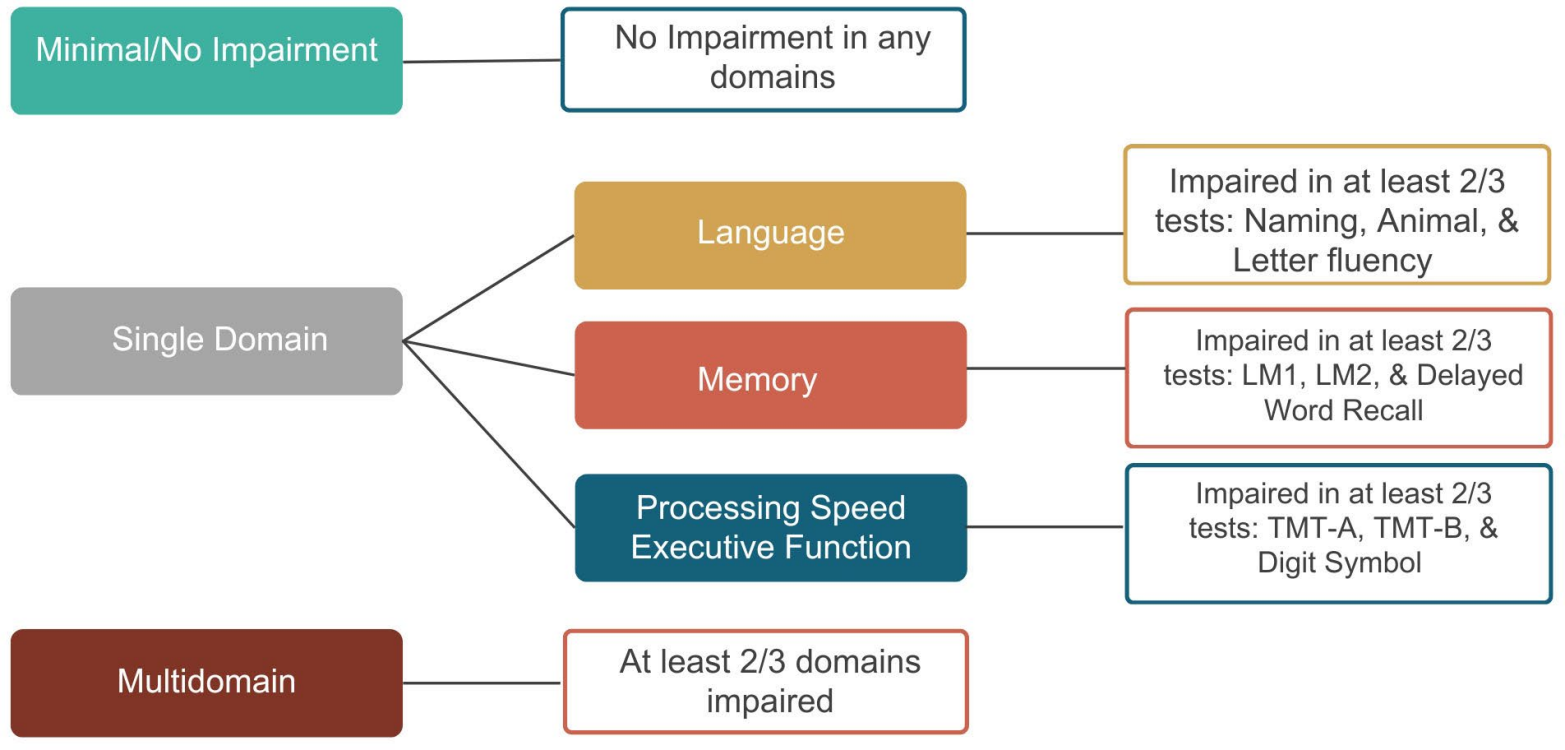
Diagnostic classification of cognitive phenotypes. BNT, Boston Naming Test; WFT, Word Fluency Test; LM1, Logical Memory 1; LM2, Logical Memory 2; DWRT, Delayed Word Recall Test; TMT-A, Trail Making Test-A; TMT-B, Trail Making Test-B; DSST, Digit Symbol Substitution Test.

Cognitive measures were divided into three domains: language, memory, and executive function/processing speed. Figure 2 shows the phenotype classification. Unlike the ARIC diagnostic classification system (described above) which considers change in performance and functional decline in MCI classification, the phenotype classification system is based on cognitive test performance only to allow for the evaluation of profiles and single versus multidomain domain involvement. To be impaired in a domain, at least two tests per domain had to meet the ≤ −1.0SD cutoff. The total number of impaired domains was used to characterize the cognitive phenotypes. Participants were classified as having a multidomain phenotype if at least two out of the three domains were impaired; Single-Domain phenotype was characterized as having one impaired domain; and Minimal/No Impairment was characterized as having no domains impaired.

### 2.9. Longitudinal changes in phenotype membership

The median follow-up time between Visits 5 and 6 was 4 years. There were no differences in the timing of follow up across the cognitive phenotypes F (2,22) = 0.282, *p* = 0.757. Twenty-five of the LOE participants with Visit 5 data also had cognitive data at Visit 6 that allowed for longitudinal phenotype characterization. *Z*-scores were also calculated based on the data from Non-LOE normal participants from Visit 5 using methods described above and the cognitive phenotypes were also derived to determine changes in phenotype membership over time. A change in classification was defined as progression to a more impaired phenotype (e.g., from Minimal/No Impairment to Single Domain or Multidomain) or worsening of an already impaired phenotype (e.g., from Single Domain to Multidomain); stable was defined as no change in phenotype membership; and revert was defined as a change to a less impaired phenotype (e.g., Single Domain to Minimal/No Impairment).

### 2.10. Statistical analyses

Statistical analyses were performed using IBM SPSS Statistics (Version 28). A two-sided corrected value of p of 0.05 was considered statistically significant. Analysis of variance (ANOVA), Fisher–Freeman–Halton exact tests (FE tests), Chi-square tests, and Mann Whitney U tests were used to test for differences in clinical and demographic variables and neuropsychological performance for continuous and categorical variables. When results from the ANOVA were significant, group contrasts were assessed using *post hoc* pairwise tests with Bonferroni correction. Multiple comparisons were corrected using Benjamini-Hochberg false discovery rate for all other statistical tests. For LOE participants with seizure onset prior to cognitive testing, we ran Spearman rho correlations to examine the relationship between age of seizure onset and cognitive performance at Visit 5. Age at Visit 5 testing was first regressed from the cognitive scores and the unstandardized residuals were used in these correlation analyses.

## 3. Results

### 3.1. Clinical and demographic characteristics of LOE participants

Ninety-one LOE participants were included from Visit 5 in the final sample. Demographic and clinical characteristics for the LOE and the Non-LOE normative samples are presented in Table 1. At Visit 5, participants ranged in age from 68 to 88 years, with approximately half of the sample being female and having education greater than a high school degree. More than half of the sample met ARIC normal diagnostic criteria and 95% of the sample had at least one vascular risk factor with hypertension being the most common vascular risk factor; 18 participants had history of stroke. Approximately 33% (*n* = 30) completed Visit 5 after the onset of seizures, and the remainder 67% (*n* = 61) before the first seizure-related code. The average number of years between first seizure and Visit 5 date was 6.44 (SD = 5.22) for those with a seizure onset prior to Visit 5, and 4.52 (SD = 1.36) for those with an onset after Visit 5. There was no statistical difference in age [*t* (89) = 1.65, *p* = 0.102; Onset before Visit 5 age mean = 78.9, Onset after Visit 5 age mean = 76.95], sex (FE value of *p* = 0.824; Onset before Visit 5 = 50% female, Onset after Visit 5 = 54.1%), or education (FE value of p = 0.824; Onset before Visit 5 = 56.7% education > high school, Onset after Visit 5 = 52.5%) between LOE participants with seizure onset prior to Visit 5 testing and those with onset after testing.

**Table 1:** Demographics and clinical variables in all LOE sample and normative sample.

	All LOE	Normative sample	Statistic	*p*-value
*N*	91	2,954		
Age	77.59 (5.34)	75.28 (4.98)	**4.35**	**<0.001**
Age at first seizure	79.40 (6.59)	–	–	–
LOE diagnosis before V5	30 (33%)	–	–	–
Sex: Female (%)	48 (52.7%)	1767 (59.8%)	–	0.193
Race: Black (%)	28 (30.8%)	564 (19.1%)	–	**<0.001**
Education: >HS	49 (53.8%)	1,298 (44.1%)		0.068
Occupational attainment: High | Low	45 (57%) | 34 (43%)	1,098 (44.8%) | 1,353 (55.2%)	–	**0.038**
ARIC cognitive diagnosis: Normal | MCI | Dementia	48 (52.7%) | 29 (31.9%) | 14 (15.4%)	–	–	–
Hypertension (Visit 5)	74 (82.2%)	2,155 (73.5%)	–	0.091
Diabetes (Visit 5)	36 (39.6%)	731 (25.5%)	–	**0.002**
Hyperlipidemia (Visit 5)	26 (28.6%)	2,924 (99%)	–	**<0.001**
BMI ≥ 30 (Visit 5)	20 (22%)	1,015 (34.4%)	–	**0.013**
Stroke*	18 (19.8%)	–		
Smoking: Current | Former | Never | Not Reported	4 (4.5%) | 48 (54.5%) | 27 (30.7%) | 9 (10.2%)	155 (5.4%) | 1,391 (48.3%) | 1,126 (39.1%) | 207 (7.2%)	*Χ*^2^ = 3.44	0.329
Alcohol Use: Current | Former | Never	37 (44%) | 30 (35.7%) | 17 (20.2%)	1,534 (54.1%) | 781 (27.6%) | 519 (18.3%)	*Χ*^2^ = 3.66	0.161
Vascular Risk Burden: 0 factors | 1 factor | 2+ factors	5 (5.6%) | 33 (36.7%) | 52 (57.8%)	2 (<1%)| 504 (17.1%) | 2,448 (82.9%)	*Χ*^2^ = 140.1	**<0.001**
APOE4 genotype: 0 allele | 1 allele | 2 alleles	44 (57.9%) | 29 (38.2%) | 3 (3.9%)	2,199 (74.4%) | 39 (1.3%) | 716 (24.2%)	*Χ*^2^ = 463.2	**<0.001**
APOE4 genotype: present	32 (42.1%)	755 (25.6%)	–	**<0.001**

^*^Non-LOE normal participants with a history of stroke were excluded in the current study. LOE, late-onset epilepsy; HS, High school; MCI, mild cognitive impairment; BMI, body mass index; High occupation complexity: managerial and professional specialty, technical, sales, and administrative support; Low occupation complexity: service, precision production, repair, operators, fabricators, laborers, homemakers; Vascular risk burden: number of vascular risk factors present including hypertension, diabetes, hyperlipidemia, obesity, and self-reported smoking.

Bold values means significant with an False Discovery Rate (FDR) correction.

### 3.2. Z-score calculation

A total of 2,954 participants were classified as Non-LOE Normal Participants (Non-LOE NP; Black = 564; White = 2,391). Given that *z*-scores were calculated separately for each racial group, demographic and cognitive data are stratified by race. Supplementary Table S2 shows demographic variables and average cognitive scores across the neuropsychological measures and Supplementary Table S3 shows comparisons of demographic variables between Non-LOE and LOE participants. There were differences in age between both sets of groups, with the LOE participants being older on average (White LOE = 77.52, Black LOE = 78.13 years versus White Non-LOE = 75.47, Black Non-LOE: 74.46 years). There were no differences in sex or education between the groups.

### 3.3. Relationship between seizure onset and cognitive performance

**Figure 3:**
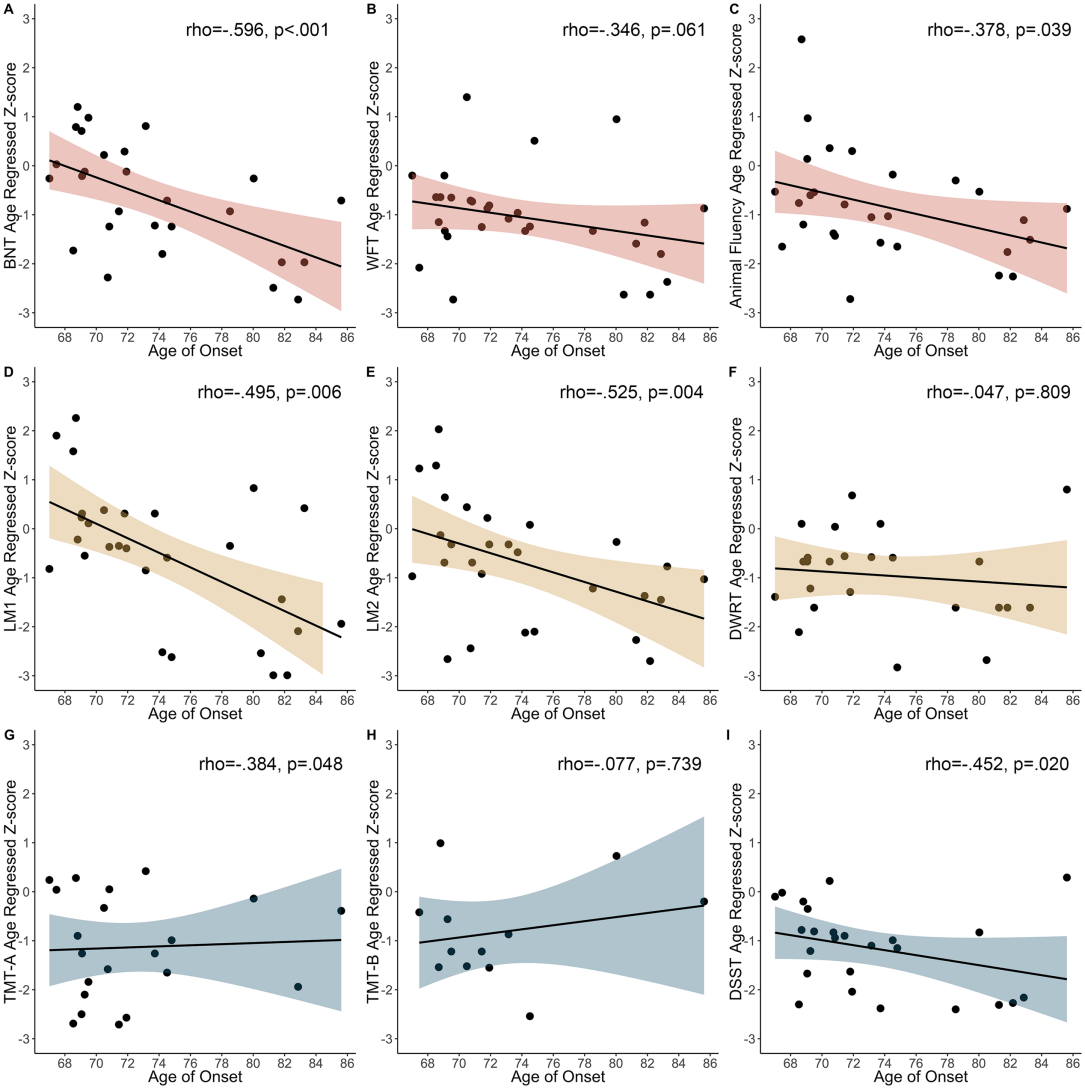
Relationship between age of first seizure and age regressed z-scores in participants with seizure onset prior to cognitive testing. **(A)** BNT, Boston Naming Test. **(B)** WFT, Word Fluency Test. **(C)** Animal Fluency. **(D)** LM1, Logical Memory 1. **(E)** LM2, Logical Memory 2. **(F)** DWRT, Delayed Word Recall Test. **(G)** TMT-A, Trail Making Test-A. **(H)** TMT-B, Trail Making Test-B. **(I)** DSST, Digit Symbol Substitution Test.

We observed an inverse relationship between the BNT (rho = −0.596, *p* < 0.001), Animal fluency (rho = −0.378, *p* = 0.039), LM1 (rho = −0.495, *p* = 0.006), LM2 (*r* = −0.525, *p* = 0.004), TMT-A (rho = −0.384, *p* = 0.048), and DSST (rho = −0.452, *p* = 0.020), with an older age of seizure onset associated with worse cognitive performance (Figure 3). There were no other significant correlations (WFT rho = −0.346, *p* = 0.061; DWRT rho = −0.047, *p* = 0.809; TMT-B rho = −0.077, *p* = 0.739).

### 3.4. Rates of impairment in LOE

**Figure 4:**
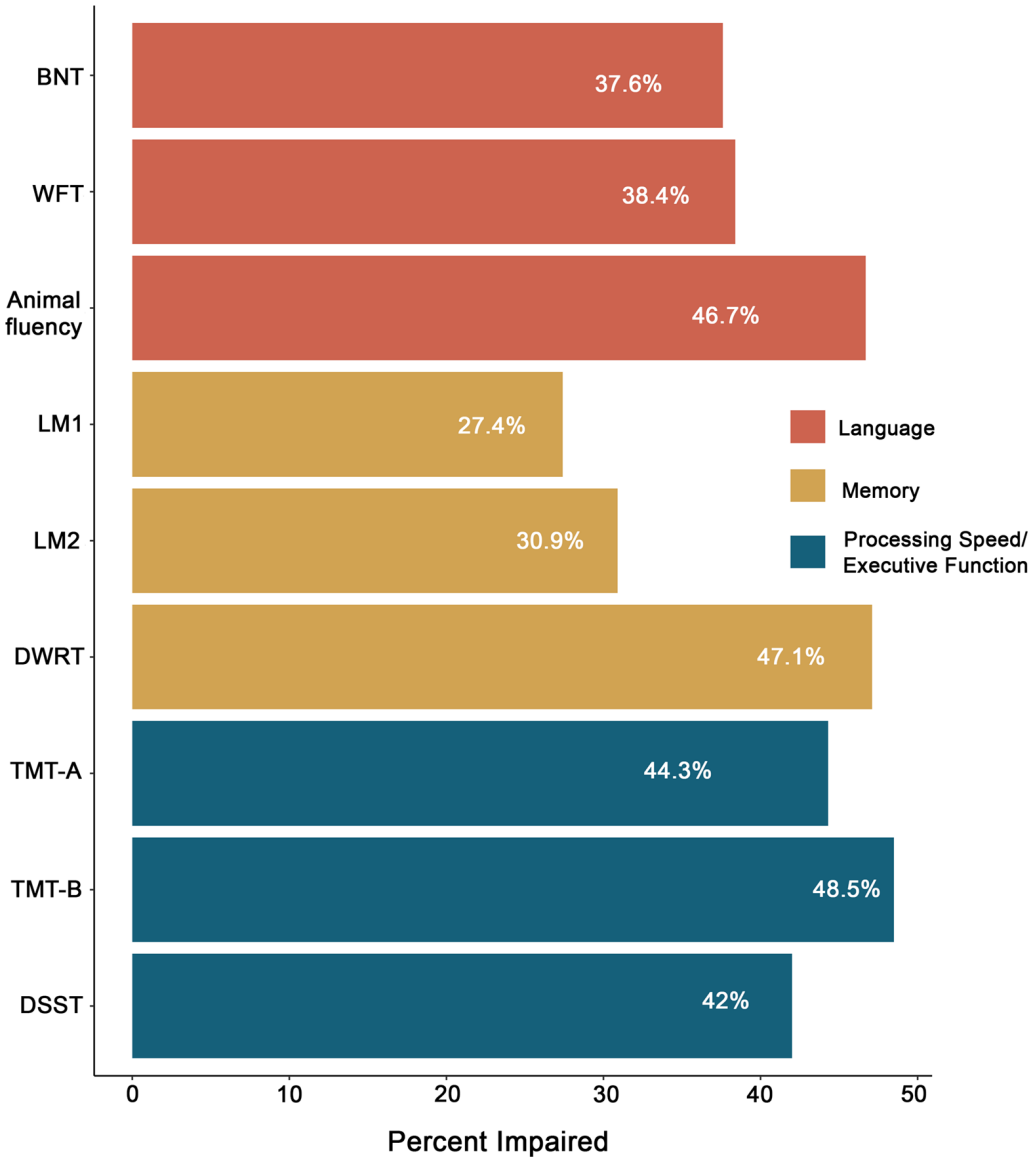
Percentage of impairment in LOE across neuropsychological measures based on a ≤ −1.0SD cutoff. BNT, Boston Naming Test; WFT, Word Fluency Test; LM1, Logical Memory 1; LM2, Logical Memory 2; DWRT, Delayed Word Recall Test; TMT-A, Trail Making Test-A; TMT-B, Trail Making Test-B; DSST, Digit Symbol Substitution Test.

Figure 4 demonstrates the pattern of impairment across individual measures at Visit 5 using the ≤ −1.0SD cutoff. Rates of impairment in language ranged from 37.6% (BNT) to 46.7% (Animal fluency); impairment rates in memory ranged from 27.4% (LM1) to 47.1% (DWRT); and impairments rates in executive function/processing speed ranged from 44.3% (TMT-A) to 48.5% (TMT-B). At the domain level (i.e., two impaired measures within a domain), 39.8% of the total sample was impaired in language, 39.5% in executive function/processing speed, and 29.3% in memory.

### 3.5. Differences in cognitive performance between participants with seizure onset prior to and after cognitive testing

Differences in performance between those with seizure onset prior to and after V5 was significant for WFT [t (84)= 3.04, *p* = 0.003; before V5 mean z-score = −1.05; after V5 mean = −0.331], TMT-A [t (77)=2.07, *p* = 0.042; before V5 mean z-score = −3.24; after V5 mean = −1.26], TMT-B [t (64)=2.89, *p* = 0.005; before V5 mean z-score = −2.32; after V5 mean = −0.976], and Digit Symbol [t (79)=2.23, *p* = 0.029; before V5 mean z-score = −1.25; after V5 mean = −0.700] with those with seizure onset prior to V5 having lower scores. Rates of impairment between the groups differ for TMT-A (*p* = 0.019; Before V5 63% impaired versus 34.6%) and TMT-B (*p* = 0.017; Before V5 71.4% impaired versus 37.8%).

### 3.6. Cognitive phenotypes in LOE

**Figure 5:**
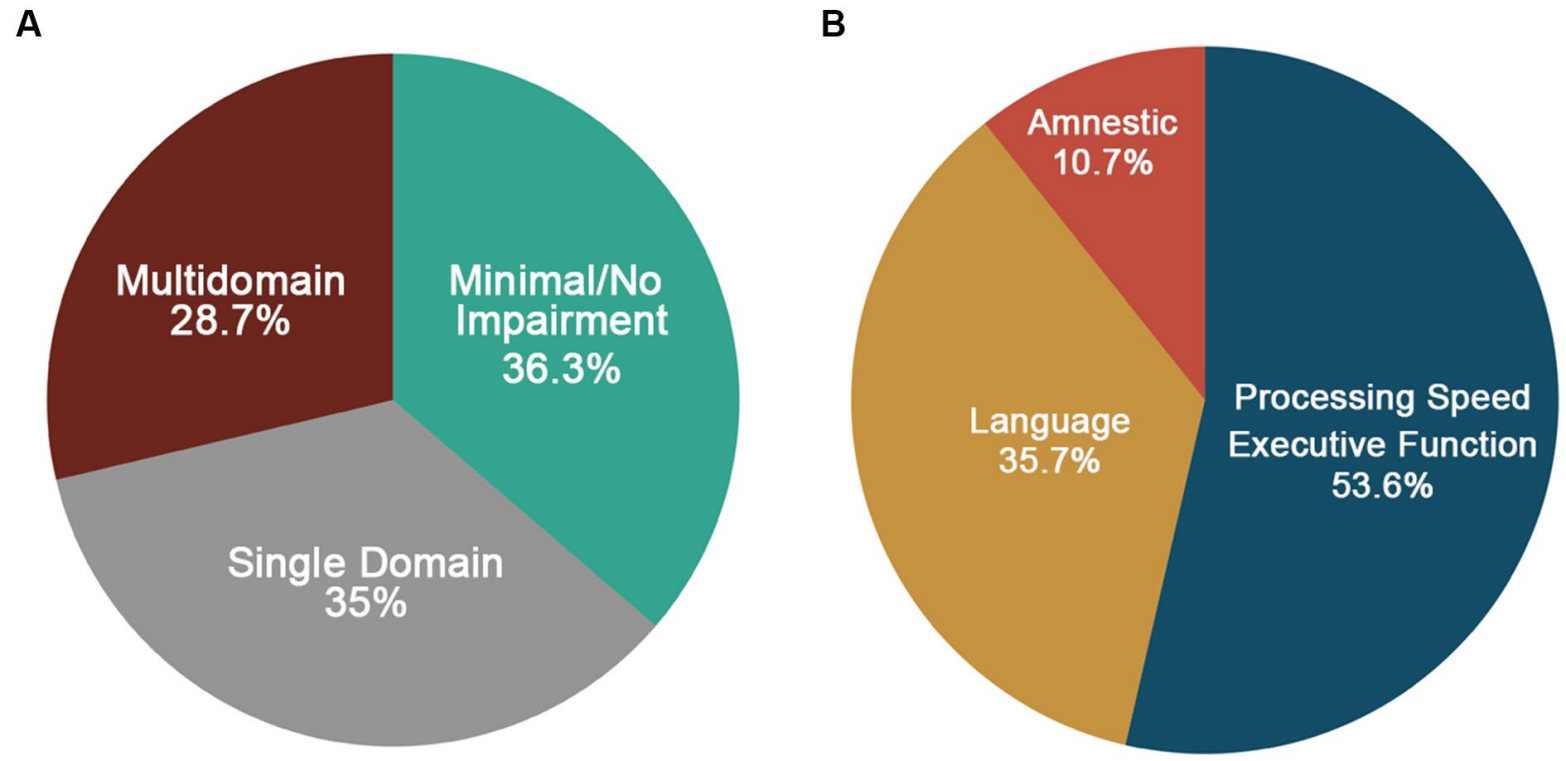
**(A)** Distribution of cognitive phenotypes in LOE based on a ≤ −1.0SD cutoff. **(B)** Distribution of domains impaired for the single domain phenotype.

**Figure 6:**
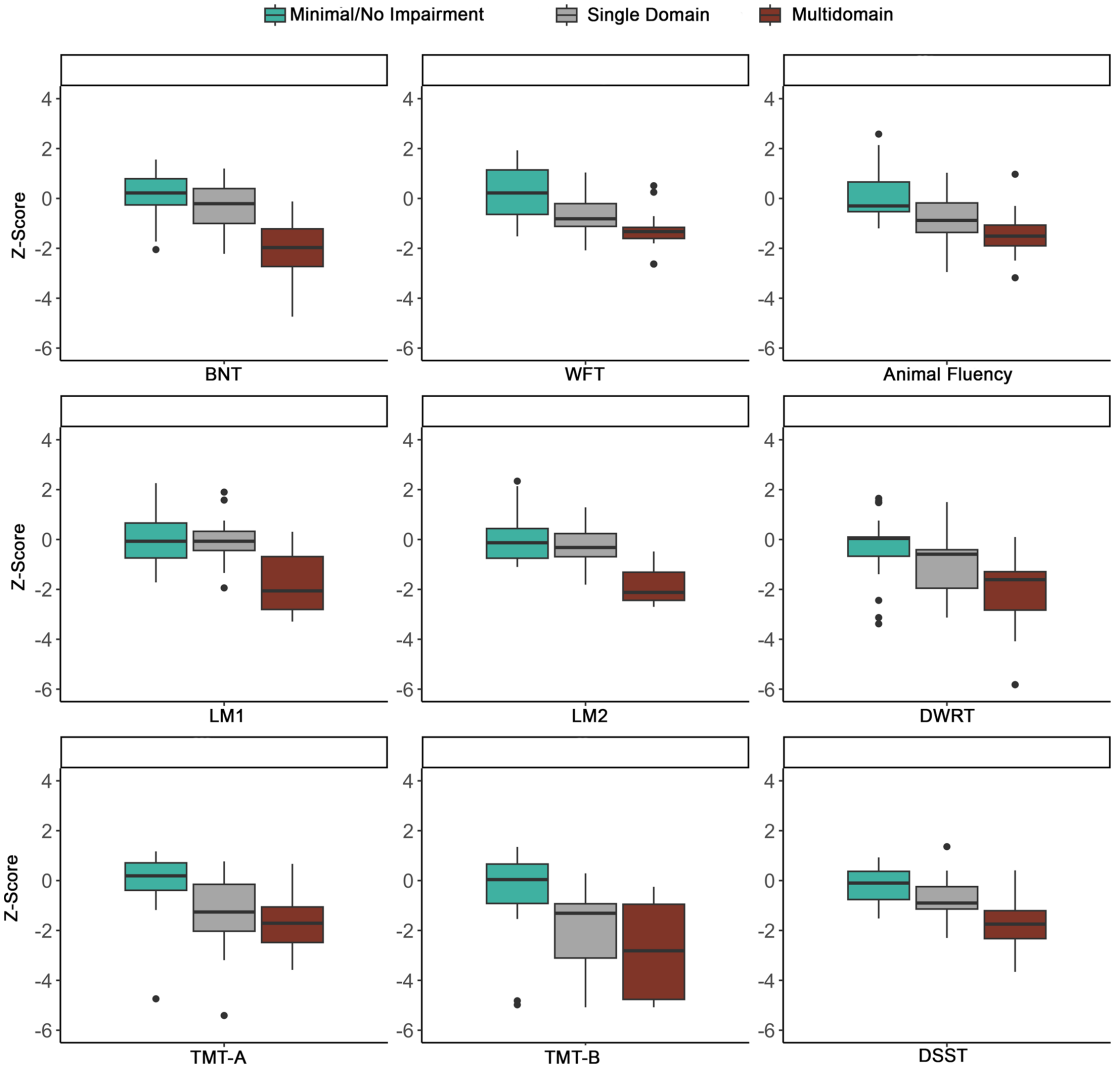
Distribution of *z*-scores across phenotypes using the ≤ −1.0SD cutoff. To show the distribution across tests, extreme outliers were removed. BNT, Boston Naming Test; WFT, Word Fluency Test; LM1, Logical Memory 1; LM2, Logical Memory 2; DWRT, Delayed Word Recall Test; TMT-A, Trail Making Test-A; TMT-B, Trail Making Test-B; DSST, Digit Symbol Substitution Test.

Of the 91 participants, 80 had complete cognitive data and were included in the phenotyping classification. Figure 5A shows the distribution of cognitive phenotypes. Twenty-seven and a half percent of the final LOE sample demonstrated a Multidomain impaired phenotype with impairments in at least two out of three cognitive domains. Within the Multidomain phenotype, 65.2% of participants had impairment in two domains with 53.3% of these participants having impairments in Language and Memory and the remainder 46.7% impairments in executive function/processing speed plus another domain. Thirty-five percent demonstrated a Single-Domain phenotype with 53.6% showing deficits in executive function/processing speed, 35.7% demonstrating language impairments and 10.7% an amnestic profile (i.e., isolated impairments in memory) (Figure 5B). Thirty-six and three tenths demonstrated a Minimal/No Impairment profile with 37.9% of the group showing no impairment in any of the tests and the remainder of the group having impairment on at least one test (34.5% one test, 20.7% two tests, and 6.9% three tests). Figure 6 shows the distribution of z-scores at the individual test level for each cognitive phenotype. Supplementary Table S4 includes average z-scores for each test across the cognitive phenotypes and group comparisons.

The distribution of cognitive phenotypes was different between participants that developed seizures prior to and after Visit 5 cognitive testing (FE = 6.61, *p* = 0.037; Onset before Visit 5: Multidomain = 39.3%, Single = 42.9%, Minimal/No Impairment = 17.9%; Onset after Visit 5: Multidomain = 23.1%, Single = 30.8%, Minimal/No Impairment = 46.2%), with participants with onset after Visit 5 having a greater proportion of participants with Minimal/No Impairment. The majority of the participants in the Single Domain phenotype had impairments in executive function/processing speed for both groups (Onset before Visit 5: 66.7%; Onset after Visit 5: 43.8%). The distribution of phenotypes without participants with dementia based on the ARIC definition were as followed: Multidomain = 23.6%, Single = 37.5%, and Minimal/No Impairment = 38.9%.

Lastly, we conducted a post-hoc sensitivity analysis to determine the rates of impairment within the Non-LOE normal control sample. We selected 10% of the normal sample and calculated z-scores based on the remainder 90% of the sample. We applied the same phenotype classification described above. The majority of this subsample demonstrated a Minimal/No Impairment profile (78.3%), followed by Single Domain (16.5%) and Multidomain (5.2%). This distribution was significantly different from the LOE phenotype distribution (*χ*^2^ = 54.17, *p* < 0.001).

### 3.7. Demographic and clinical variables across phenotypes

Table 2 includes demographic and clinical factors across cognitive phenotypes. There were differences in age, with the Multidomain phenotype being older compared to the Single Domain phenotype (81.13 years versus 75.54 years, *p* < 0.001) and Minimal/No Impairment (76 years, *p* = 0.001). There were differences in age of seizure onset, with the Multidomain phenotype having an older age of seizure onset compared to the Single Domain phenotype (81.7 years versus 75.92, *p* = 0.005) and there was a trend toward an older age of seizure onset compared to the Minimal/No Impairment (79.89 years, *p* = 0.058). There were differences in education with participants with the Multidomain phenotype having a lower proportion of older adults (26.1%) with an education higher than a high school degree compared to the Single Domain (46.4%) and Minimal/No Impairment (79.3%). Lastly, there were differences in occupational attainment with participants with the Multidomain phenotype having lower occupational complexity relative to the other two groups. Although there were no differences in vascular risk factors across the phenotypes, hypertension was the most common factor with 85.7% of the Single Domain, 78.3% of the Multidomain, and 75.9% of the Minimal/No Impairment phenotypes having hypertension. There were no other differences across phenotype groups.

**Table 2:** Clinical and demographic characteristics across LOE cognitive phenotypes.

	Multidomain	Single-domain	Minimal	*F*	*p*-value
*n* (%)*	23 (28.7%)	28 (35%)	29 (36.3%)		
**Age**	**81.1 (4.76)**	**75.5 (5.54)**	**76.0 (4.36)**	**9.82**	**<0.001**
Age at 1^st^ seizure	**81.71 (6.73)**	**75.92 (5.95)**	**79.89 (6.22)**	**5.79**	**0.005**
				FE	*p*-value
Sex: Female	14 (60.9%)	13 (46.4%)	16 (55.2%)	1.10	0.59
Race: Black	9 (39.1%)	7 (25%)	7 (24.1%)	1.66	0.45
**Education:** >**HS**	**6 (26.1%)**	**13 (46.4%)**	**23 (79.3%)**	**15.5**	**<0.001**
**Occupational complexity: High**	**5 (27.8%)**	**16 (64%)**	**18 (72%)**	**8.83**	**0.008**
Antiseizure medications: Yes	1 (4.3%)	0 (0%)	1 (3.4%)	–	–
Stroke	7 (30.4%)	4 (14.3%)	7 (24.1%)	1.99	0.39
Hypertension	18 (78.3%)	24 (85.7%)	22 (75.9%)	0.980	0.69
Diabetes	10 (43.5%)	13 (46.4%)	10 (34.5%)	0.936	0.66
BMI ≥ 30	5 (21.7%)	10 (35.7%)	10 (34.5%)	1.38	0.52
Hyperlipidemia	5 (21.7%)	6 (21.4%)	8 (27.6%)	0.406	0.85
Vascular burden: 2 or more	13 (56.5%)	19 (67.9%)	16 (55.2%)	1.15	0.60
APOE4 genotype: Present	8 (36.4%)	8 (38.1%)	10 (41.7%)	0.197	0.95

^*^Based on the total number of participants with complete cognitive data. LOE, late-onset epilepsy; HS, High school; BMI, body mass index; FE, Fisher–Freeman–Halton exact test. High occupation attainment: managerial and professional specialty, technical, sales, and administrative support. Low occupation attainment: service, precision production, repair, operators, fabricators, laborers, homemakers. Bold: significant with an FDR correction of 0.01.

Seventy-five percent (*n* = 6) of the participants with an ARIC dementia diagnosis demonstrated a Multidomain phenotype, while the remainder two participants had Single Domain or Minimal/No Impairment phenotype. Of those with MCI, 61.5% had a Single phenotype, 26.9% Multidomain, and 11.4% a Minimal/No Impairment phenotype. In those with a Normal Cognition diagnosis based on the ARIC definition, 54.3% had a Minimal/No Impairment phenotype, 23.9% Single Domain, and 21.7% a Multidomain phenotype.

### 3.8. Longitudinal changes in phenotype membership

At ARIC-NCS Visit 6, 25 (27.5%) participants completed testing, 23 (25.3%) were deceased, and the remainder 43 (47.3%) did not complete Visit 6. Out of the 25 participants with LOE and longitudinal data, 12 had a Minimal/No Impairment and 12 had a Single Domain phenotype at Visit 5. The one participant that had a Multidomain phenotype at Visit 5, remained stable at Visit 6 and was not included in additional analyses. Of these 24 participants, 62.5% declined and 37.5% remained stable; no participants reverted. Of those that declined, 46.7% had a Minimal/No Impairment phenotype at Visit 5. Of those that remained stable, 44.4% had a Single Domain and 55.6% a Minimal/No Impairment phenotype. When comparing demographic and clinical variables between the participants that declined and those that remained stable, the participants that remained stable had higher levels of education (Table 3). Out of the 15 individuals that declined, 3 (20%) met criteria for a new diagnosis of MCI and 3 (20%) a diagnosis of dementia based on the ARIC diagnostic definitions. For those that decline at Visit 6, executive function (50%) was the most commonly impaired domain at Visit 5, followed by language (37.5%), and memory (12.5%).

**Table 3:** Clinical and demographic characteristics between stabled and declined participants.

	Stabled	Decline	Comparison
*n*	9 (37.5%)	15 (62.5%)	
Age (V5)	75.56 (3.84)	74.53 (4.88)	U = 67, *p* = 1.00
Age at 1^st^ seizure	75.41 (5.83)	77.53 (4.89)	U = 84, *p* = 0.35
Sex: F (%)	3 (33.3%)	8 (53.3%)	U = 0.427, *p* = 0.69
Race: Black (%)	1 (11.1%)	5 (33.3%)	FE = 0.906, *p* = 0.42
**Education: > HS**	**9 (100%)**	**6 (40%)**	**FE = 8.64, *p* = 0.007**
Vascular Risk (1+ factors)	9 (100%)	13 (86.7%)	FE = 1.31, *p* = 0.51
APOE4 Present	3 (50%)	3 (25%)	FE = 1.25, *p* = 0.34

HS, high school. Bold: significant at *p* = 0.05.

## 4. Discussion

With a globally aging population and the expected increase in the number of individuals with LOE, it is important to fully characterize the cognitive profiles of older adults with epilepsy to identify those at increased risk for progressive cognitive decline. Here, we show that approximately 63% of older adults who developed LOE demonstrate an impaired cognitive phenotype (i.e., Multidomain or Single Domain phenotype) and that in a sizable subset of individuals, an impaired profile is present prior to the onset of recognized seizures. Further, executive function/processing speed was the most impaired domain in those with isolated impairment and for those patients that declined. We also show that more than half of the participants with longitudinal cognitive data progressed to a more impaired phenotype. Lastly, higher education was associated with minimal or no impairment at our baseline visit (Visit 5) and a lower likelihood of declining over time.

### 4.1. Cognitive impairment in LOE

Given that the small number of studies that have exclusively focused on LOE have included cognitive screeners or had a limited number of neuropsychological tests (22–24, 35–39), the full characterization of cognitive profiles in LOE remains to be examined. In this study, we show that in a population of older adults with LOE who completed a cross-sectional assessment, cognitive impairment is common across a comprehensive battery of tests with rates of impairment ranging from 27% to 48%, with measures of set-shifting, delayed recall, semantic fluency, and processing speed the most prevalent. At the domain level, more than a third of the sample was impaired on at least one domain despite our rather stringent criteria, which required two out of the three measures per domain to be impaired; an approach which has been shown to provide a good balance between sensitivity and specificity for classifying impairment in older adults and which may explain its diagnostic stability across cohorts (71).

In our study, participants with impairments included those with an onset of seizures either prior to or after Visit 5 cognitive testing, and with more than half the sample demonstrating an impaired phenotype. Studies in LOE have reported poorer cognitive outcomes that in some patients are present before the onset of seizures (24, 31, 38). In a larger sample of older adults with LOE from the ARIC study (24), we previously showed that a steeper longitudinal decline in cognitive function occurred prior to the onset of seizures in those who developed LOE versus those who did not, and that this decline in LOE became more rapid after the onset of seizures. Our results support these earlier findings in that in a subset of patients who develop LOE, cognitive dysfunction is present before the onset of seizures with some evidence of progression over time. Noteworthy, the presence of cognitive impairment at the time of an epilepsy diagnosis has been documented across several studies (36, 39, 72–75) implying that cognitive dysfunction may not always be solely caused by the accumulating effects of seizures or the long-term exposure to epilepsy treatment (e.g., antiseizure medications, surgery) but rather may be a result of epileptogenesis or other etiological factors that contribute to the development of seizures later in life (e.g., small vessel disease).

We also show that in those with a seizure onset prior to cognitive testing, an older age of seizure onset was associated with poorer performance on measures of naming, verbal fluency, learning and memory, and processing speed. In contrast, a younger age of seizure onset has been linked to an increased risk for cognitive impairment in early onset epilepsy (40). Thus, developing seizures in older age may accelerate cognitive decline. Interestingly, Liguori and colleagues (38) demonstrated that in a group of older adults with LOE cognitive progression on a global measure of cognitive ability was observed at a 12-month follow-up irrespective of type and number of antiseizure medications. Our findings and that of previous studies highlight that the relationship between classic epilepsy characteristics and cognition may be different for LOE and therefore, more studies are needed to better delineate these associations given their implications for treatment and long-term outcomes.

### 4.2. Cognitive phenotypes in LOE

An important advantage of the phenotyping approach is that it is patient-centered and considers the individual variability within neurological disorders. Traditional approaches to studying neuropsychological syndromes aggregate patients based on the neurological condition (e.g., all patients with epilepsy); however, this approach may obscure important differences across syndrome/disorder subtypes. Further, the phenotype classification reflects the process that clinicians employ which typically consists of examining multiple scores within a domain and base clinical decisions on the pattern of scores rather than isolated impaired scores (71). In the MCI literature, the prodromal phase of AD, identifying cognitive profiles or phenotypes has proven useful for predicting cognitive decline and progression to dementia (42, 76). Specifically, an amnestic, dysnomic, and dysexecutive/mixed phenotypes have been described with unique MRI signatures and differential progression to dementia (41, 42, 76). In our study, a majority of the participants with a dementia diagnosis based on the ARIC definition had a Multidomain phenotype (75%), while those with MCI had a Single Domain (61.5%) phenotype. Although for a large proportion of the participants the pattern of impairment (i.e., phenotype) matched the severity of the diagnosis (e.g., multidomain impairment and a dementia diagnosis), this was not the case for all participants. Given that phenotypes are based on the pattern of cognitive scores alone and do not take into account changes in functional activities of daily living, some individuals may demonstrate a less impaired phenotype but greater functional decline and therefore may meet criteria for dementia. Thus, cognitive phenotypes do not replace diagnostic criteria for MCI and dementia, rather, they help characterize the underlying cognitive profiles within these diagnostic categories. Specifically, an MCI or dementia diagnosis provides information on whether an individual has significant cognitive impairments or has declined from a previous level of functioning, whereas cognitive phenotypes delineate the different patterns of impairment. Therefore, our study provides additional information on the cognitive subtypes associated with LOE, which, when considered in combination with other disease biomarkers, may shed light on differential risk for further cognitive decline.

In our sample, approximately 29% of the older adults demonstrated *global* or Multidomain impairment, which is comparable to rates reported in studies of young-to-middle-aged adults with temporal (40, 50, 51, 53–55) and frontal lobe epilepsy (52). Patients with global impairments are thought to represent a group of patients with potential co-morbid non-epilepsy pathology, elevated health-related risk factors, or sociodemographic factors that may be resulting in greater cognitive dysfunction than expected. Furthermore, these patients demonstrate widespread brain abnormalities that extend beyond the seizure focus potentially explaining the multidomain (i.e., multi lobar) involvement in this impaired profile (40, 49, 51). In our study, individuals with a Multidomain phenotype were older, had fewer years of education, and lower occupational complexity. Fewer years of education has been a consistent finding in patients with epilepsy demonstrating global impairment (40). Thus, these factors may be contributing to their global impaired profile by further exacerbating the effects of epilepsy pathology on cognition. Another possibility is that for some individuals these cognitive deficits were longstanding and therefore may have led to lower educational and occupational attainment. For example, a preexisting learning disability in early childhood may have impacted a person’s educational attainment. However, given the nature of our data, we did not have information on early history of cognitive dysfunction and how that may have impacted education/occupational attainment. Although we did not have EEG information on seizure localization/lateralization, the Multidomain phenotype in our study may represent a phenotype with widespread brain anomalies that may be associated with both epilepsy and non-epilepsy pathology.

The Single Domain phenotype was characterized by prominent impairments in executive function/processing speed. By contrast, in younger adults with frontal lobe epilepsy (52) and temporal lobe epilepsy (53), the Single Domain phenotype has been characterized by impairments in language with naming and verbal fluency the most impaired cognitive processes. In fact, in a sample of 1,409 young-to-middle aged adults with temporal lobe epilepsy, 49% of the patients with a Single Domain phenotype had isolated deficits in language (53). In a sample of Spanish-speaking patients with temporal lobe epilepsy, memory was the most commonly impaired domain within the Single Domain phenotype (55). The differences in the nature of the Single Domain phenotype across studies may be due to varying underlying epilepsy etiologies, epilepsy-related clinical factors, sociodemographic characteristics, or differences in brain abnormalities (e.g., lateral versus mesial temporal lobe involvement). Given the nature of epilepsy ascertainment in ARIC, we did not have comprehensive clinical information to examine the epilepsy characteristics associated with these isolated impairments in executive function/processing speed such as brain pathology involving the frontal lobes. However, the high vascular burden in our overall sample may be a contributing factor. Although there were no differences in the number and type of vascular risk factors across the phenotypes, approximately 95% of the sample had at least one vascular risk factor with hypertension being the most common. Elevated vascular risk factors have been associated with the extent of cognitive impairment in patients with epilepsy (77, 78) and with an increased risk of developing LOE (8). In the general population, vascular risk factors, particularly diabetes and hypertension are associated with cognitive decline and dementia (79). Specifically, the presence of vascular risk factors has been implicated in executive dysfunction and slower processing speed given their effects on white matter structures involved in these domains (80, 81). We previously demonstrated an association between increased white matter hyperintensities burden and increased likelihood of developing LOE in a larger sample of participants from ARIC which includes the sample in the current study (61). Given that LOE may be associated with varying etiologies, an executive dysfunction and reduced processing speed phenotype/profile may be indicative of the presence of occult cerebrovascular disease. Notably, impairments in executive function and processing speed (82) may reflect vascular involvement and thus it is possible that a subset of older adults with a dysexecutive and slowed processing speed profile may be at risk for the development of dementia of a mixed or vascular etiology. However, given the small number of LOE participants with dementia in our study, we were not able to delineate differences in profiles.

Lastly, 36.3% of the sample demonstrated a Minimal/No Impairment phenotype which included a subset of participants (37.9%) with no impairment in any of the tests and the majority demonstrating 1–2 impaired scores. Notably, studies have shown that the vast majority of adults demonstrate 1–2 impaired performances across a larger neuropsychological battery (83–85). Thus, an advantage of the phenotype approach is reducing the likelihood of false positives that may result when examining individual test scores in isolation. The Minimal/No Impairment phenotype in our study was characterized by higher education with more than 65% of the older adults having an advanced degree (i.e., college or graduate degree). Across studies, the rates of this phenotype/profile have ranged from 16% to 54% and have been associated with less disease burden including shorter disease duration, fewer antiseizure medications, and less brain pathology (40). Further, higher education in this group has been a consistent finding across investigations. Higher levels of education and complex occupational attainment have been hypothesized to increase cognitive reserve, a protective mechanism that mitigates the effects of brain pathology on cognition by increasing the cognitive resources available to compensate for cognitive deficiencies. For example, higher levels of education have been associated with a lower risk of developing dementia and/or a delay in the onset of dementia-related symptoms. (86–88). In epilepsy, higher levels of education have been shown to protect against the effects of epilepsy related pathology on cognition, as patients with higher education demonstrate less cognitive impairments despite showing greater disease burden (40, 89). Studies with larger samples examining the clinical and demographic profiles of patients with minimal impairment can help identify protective factors which can inform clinical interventions aimed are reducing cognitive decline.

### 4.3. Cognitive progression

Identifying distinct cognitive phenotypes has been shown to be useful in predicting cognitive progression. In the subset of our sample with longitudinal data, we show that 62.5% of the older adults with either a Single Domain or a Minimal/No phenotype decline (i.e., changed to a more impaired phenotype) at a subsequent visit. Fifty percent of those that decline demonstrated an executive dysfunction profile at baseline and although we were not able to statistically evaluate its predicted value, executive function deficits may be associated with cognitive progression, potentially due to a vascular underlying etiology. Whether epilepsy results in accelerated brain and cognitive aging has been an ongoing debate in the literature. Studies have provided evidence of brain aging in patients with epilepsy that includes both patients with long-standing and newly onset epilepsy (90–92). Importantly, there is evidence of cognitive deterioration regardless of the age of onset (24, 38, 93, 94). In the MCI/AD literature, phenotypes have been shown to have prognostic value improving prediction of clinical course (41). Thus, phenotyping may provide a promising approach to stratifying risk for decline that considers individual variability within patient cohorts and could help identify factors that constitute this group and may buffer against decline (e.g., education).

### 4.4. Limitations

There are several limitations to our study that limit the generalizability of the findings. First, the use of ICD codes to diagnose epilepsy can potentially lead to misclassification of diagnosis. Inherent in this use of code data is the potential to miss cases of childhood epilepsy that have resolved, or to misclassify recurrent provoked seizures as epilepsy if there were multiple hospitalizations for alcohol withdrawal seizures (for example). However, the method (i.e., ≥ 2 ICD codes) used has been shown to be robust with high sensitivity and specificity (24, 60). Second, our sample size was modest compared to other studies involving cognitive phenotypes in epilepsy. Further, the lack of differences in vascular risk factors across the phenotypes may be explained by the sample size and the fact that most participants in our sample had a high vascular burden and therefore there was less heterogeneity. Studies with larger samples of older adults with LOE are needed in order to replicate our findings and to identify unique vascular and other risk factor profiles associated with each phenotype. Third, although we used a normative sample to account for the effects of age, education, sex, and race on cognitive scores, there were age differences between the normative sample size and the LOE participants. Fourth, we did not have comprehensive epilepsy-related clinical data such as seizure frequency and number of antiseizure medications and type, and therefore could not examine the relationship between phenotype membership and classic epilepsy variables (e.g., antiseizure medications, EEG findings, seizure frequency, epilepsy etiology); full, optimal workup of new-onset epilepsy including lumbar puncture was not available (if performed) (95). Importantly, better epilepsy characterization (i.e., seizure localization/lateralization based on EEG and imaging findings) can help delineate the brain regions associated with different cognitive profiles (i.e., executive function/processing speed = frontal lobe abnormalities; amnestic profile = mesial temporal lobe abnormalities). Based on evidence from several phenotype investigations, two major patterns have emerged with global deficits associated with greater disease burden and elevated risk factors for cognitive impairment while patients with relatively intact profiles demonstrating less disease burden and protective factors. However, these findings have been found primarily in patients with an earlier age of epilepsy onset that have been fully characterized and therefore studies are needed to determine the clinical profiles associated with each phenotype in LOE. Fifth, we only had longitudinal data in a subset of the sample and therefore, longitudinal studies with large samples are needed in order to determine the diagnostic value of the phenotype approach in determining risk of cognitive progression in LOE. Further, selectivity of attrition (e.g., participants returning for cognitive testing due to concerns of decline) could have introduced bias in the longitudinal sample. Interestingly, a study examining differences in cognitive abilities and personality traits between returning and non-returning participants found that returning participants demonstrated higher cognitive abilities and personality traits such as agreeableness and openness which was more apparent in adults older than 50 (96). Thus, it is possible that those participants that returned had better insight into their cognitive abilities and were worried about decline. Lastly, although we used all participants without epilepsy and with normal cognition as our normative sample for determining impairment profiles, future studies comparing the rates and patterns of cognitive impairment (i.e., phenotypes) between LOE participants and Non-LOE participants with other neurological conditions (e.g., MCI, TBI or dementia) can elucidate whether epilepsy is associated with unique patterns of cognitive impairment and/or confers a differential risk for cognitive decline beyond the effects of aging on cognition.

## 5. Conclusion

This study delineates unique cognitive phenotypes in LOE using a large, population-based study cohort. Our findings demonstrate heterogeneity in cognitive impairment within LOE that can be appreciated by identifying cognitive phenotypes. Thus, the application of this approach may accelerate our understanding of the clinical course of LOE, and guide future interventions aimed at preventing the onset of cognitive dysfunction or reducing the risk of further cognitive decline in older adults.

## Data availability statement

This study analyzed publicly available datasets. These data can be found via application for ARIC Data or through the NIH NHLBI-sponsored Biologic Specimen and Data Repository Information Coordinating Center (BioLINCC) at: https://biolincc.nhlbi.nih.gov/.

## Ethics statement

The studies involving human participants were reviewed and approved by all participating ARIC Institutions Institutional Review Boards. All participants provided written informed consent.

## Author contributions

AR: drafting/revision of the manuscript for content, study concept or design, and analysis or interpretation of data. AS, AK-N, and RG: drafting/revision of the manuscript for content and interpretation of data. EJ and CM: drafting/revision of the manuscript for content, study concept or design, and interpretation of data. All authors contributed to the article and approved the submitted version.

## Funding

The Atherosclerosis Risk in Communities Study is carried out as a collaborative study supported by National Heart, Lung, and Blood Institute contracts (75N92022D00001, 75N92022D00002, 75N92022D00003, 75N92022D00004 and 75N92022D00005). The ARIC Neurocognitive Study is supported by U01HL096812, U01HL096814, U01HL096899, U01HL096902, and U01HL096917 from the NIH (NHLBI, NINDS, NIA and NIDCD). AS was supported by the National Institute of Neurological Disorders and Stroke K23NS123340. RG was supported by the NINDS Intramural Research Program. McDonald was supported by the National Institute of Neurological Disorders and Stroke R01 NS120976. AR was supported by a Diversity Supplement (R01 NS120976), a Clinical Research Grant from the National Academy of Neuropsychology, and the Burroughs Wellcome Fund Postdoctoral Diversity Enrichment Program. EJ was supported by the National Institute of Aging K23 AG063899.

## Conflict of interest

The authors declare that the research was conducted in the absence of any commercial or financial relationships that could be construed as a potential conflict of interest.

## Publisher’s note

All claims expressed in this article are solely those of the authors and do not necessarily represent those of their affiliated organizations, or those of the publisher, the editors and the reviewers. Any product that may be evaluated in this article, or claim that may be made by its manufacturer, is not guaranteed or endorsed by the publisher.
