# Supplementary material for: Cognitive phenotypes in late-onset epilepsy: results from the atherosclerosis risk in communities study
Source: Front Neurol. 2023 Aug 24;14:1230368. doi: 10.3389/fneur.2023.1230368 (PMC10513940; doi:10.3389/fneur.2023.1230368)
Supplement: Supplementary file 1 [file Data_Sheet_1.docx]

| Supplementary Table 1: Demographic and neuropsychological scores of non-LOE normal cognition participants stratify by race | | | | |
| --- | --- | --- | --- | --- |
|  | White | Black | t | p-value |
| N | 2391 | 563 |  |  |
| Age | 75.47 (5.0) | 74.46 (4.8) | 4.48 | <.001 |
| Sex: Female | 1397 (58.4%) | 370 (65.7%) | -- | <.001 |
| Education: > HS | 1020 (42.8%) | 278 (49.6%) | -- | .002 |
| BNT | 27.43 (2.02) | 21.27 (5.71) | 24.87 | <.001 |
| WFT | 36.50 (10.9) | 31.43 (11.71) | 9.20 | <.001 |
| Animal Fluency | 17.93 (4.39) | 15.15 (4.19) | 13.53 | <.001 |
| LMI | 24.02 (6.67) | 21.17 (6.43) | 9.15 | <.001 |
| LMII | 19.5 (7.12) | 15.82 (6.67) | 11.07 | <.001 |
| DWRT | 5.88 (1.44) | 5.53 (.953) | 6.66 | <.001 |
| TMT-A* | 36.89 (10.45) | 54.61 (17.26) | 22.07 | <.001 |
| TMT-B* | 92.47 (29.52) | 162.8 (60.96) | 23.99 | <.001 |
| DSST | 43.34 (9.44) | 32.6 (10.7) | 21.64 | <.001 |
| HS: High school; BNT: Boston Naming Test; WFT: Word Fluency Test; LM: Logical Memory; DWRT: Delayed word recall test; TMT: Trail Making Test; DSST: Digit symbol substitution test  Standard deviations are represented in parenthesis  * completion time in seconds | | | | |

| Supplementary Table 2: Comparison of demographic variables between LOE and Non-LOE participants | | | |
| --- | --- | --- | --- |
|  | White Non-LOE | White LOE | Comparison |
| N | 2391 | 63 |  |
| Age | 75.47 (5.0) | 77.52 (5.11) | U= 93890, p=.002 |
| Sex: Female | 1397 (58.4%) | 36 (57.1%) | FE= .122, p=.80 |
| Education: > HS | 1020 (42.8%) | 36 (57.1%) | FE= 1.79, p=.20 |
|  | Black Non-LOE | Black LOE | Comparison |
| N | 563 | 28 |  |
| Age | 74.46 (4.82) | 78.13 (6.12) | U= 5679.5, *p*= .001 |
| Sex: Female | 370 (65.7%) | 12 (42.9%) | FE= 3.87, p=.05 |
| Education: > HS | 278 (49.6%) | 13 (46.4%) | FE= .236, p=.71 |
| LOE: late-onset epilepsy; HS: High school; FE: Fisher-Freeman-Halton exact test  Standard deviations are represented in parenthesis | | | |

| Supplementary Table 3: Comparison of cognitive scores across cognitive phenotypes | | | | | | | |
| --- | --- | --- | --- | --- | --- | --- | --- |
|  | Multidomain | Single-Domain | | Minimal |  | |  |
|  | Mean (SD) | Mean (SD) | | Mean (SD) | F | | p-value |
| **Language** | | | | | | | |
| BNT | -2.32 (2.08) | -.353 (.979) | | .084 (.876) | 22.03 | | <.001 |
| WFT | -1.35 (.783) | -.647 (.734) | | .159 (1.01) | 19.28 | | <.001 |
| Animal Fluency | -1.45 (.849) | -.793 (1.04) | | .023 (.971) | 15.80 | | <.001 |
| **Learning & Memory** | | | | | | | |
| LM1 | -1.86 (1.08) | -0.71 (.745) | | .058 (.915) | 32.09 | | <.001 |
| LM2 | -1.92 (.660) | -.178 (.721) | | .042 (.965) | 39.15 | | <.001 |
| DWRT | -2.51(1.74) | -.896 (1.102) | | -.311 (1.14) | 18.18 | | <.001 |
| **Executive Function/Processing Speed** | | | | | | | |
| TMT-A | -3.44 (4.68) | -1.37 (1.96) | | .008 (1.05) | 9.99 | | <.001 |
| TMT-B | -2.98 (2.00) | -2.14 (1.57) | | -.427 (1.50) | 12.77 | | <.001 |
| DSST | -1.79 (.906) | -.796 (.867) | | -.168 (.682) | 24.18 | | <.001 |
| ANOVA Pairwise group comparisons | | | | | | | |
|  | Multidomain vs Single-Domain | | Multidomain vs Minimal | | | Single-Domain vs Minimal | |
| BNT | <.001 | | <.001 | | | .65 | |
| WFT | .023 | | <.001 | | | .002 | |
| Animal Fluency | .06 | | <.001 | | | .006 | |
| LM1 | <.001 | | <.001 | | | 1.00 | |
| LM2 | <.001 | | <.001 | | | .93 | |
| DWRT | <.001 | | <.001 | | | .29 | |
| TMT-A | .037 | | <.001 | | | .16 | |
| TMT-B | .55 | | <.001 | | | <.001 | |
| DSST | <.001 | | <.001 | | | .014 | |
| BNT: Boston Naming Test; WFT: Word Fluency Test; LM: Logical Memory; DWRT: Delayed word recall test; TMT: Trail Making Test; DSST: Digit symbol substitution test | | | | | | | |
