# Supplementary material for: Cognitive phenotypes in late-onset epilepsy: results from the atherosclerosis risk in communities study
Source: Front Neurol. 2023 Aug 24;14:1230368. doi: 10.3389/fneur.2023.1230368 (PMC10513940; doi:10.3389/fneur.2023.1230368)
Supplement: Supplementary file 2 [file Data_Sheet_2.docx]

| Supplementary Table 1: Neuropsychological Tests | | |
| --- | --- | --- |
| Test | Description | Domain |
| Wechsler Memory Scale-Revised Logical Memory (LM) immediate (LM1) | Participants are read a short story and asked to recall the details immediately and after a 20-minute delay; each score consists of the total number of correct individual story elements recalled. | Memory |
| Delayed word recall test (DWRT) | The DWRT consists of two learning trials where participants are given 10 common nouns to learn by using each noun in a sentence, followed by a recall trial after a five-minute delay. The DWRT score is the total number of words recalled after the delay. | Memory |
| Boston Naming Test (BNT) | The BNT consists of a series of 30 line drawings and participants are instructed to name the object in each drawing; the BNT score is the total number of drawings correctly identified. | Language |
| Word fluency test (WFT) | Participants are given 60 seconds to generate as many words as possible beginning with the letters F, A, and S; WFT score is the total number of words provided across all three letters. | Language |
| Animal fluency | Participants are instructed to generate as many animals as possible in 60 seconds; score is the total number of animals provided. | Language |
| Trail Making Test Part A (TMT-A) | Participants are required to connect numbers 1-25 in sequential order; TMT-A score is the total time to completion. | Processing speed |
| Digit symbol substitution test (DSST) | Participants are asked to translate numbers to symbols based on a key; total score is the number of correctly translated symbols within 90 seconds. | Processing speed |
| Trail Making Test Part B (TMT-B) | Participants are instructed to connect circles in ascending order, alternating between numbers and letters; TMT-B score is total time to completion. | Executive Function |

| Supplementary Table 2: Demographic and neuropsychological scores of non-LOE normal cognition participants stratified by race | | | | |
| --- | --- | --- | --- | --- |
|  | White | Black | t | p-value |
| N | 2391 | 563 |  |  |
| Age | 75.47 (5.0) | 74.46 (4.8) | 4.48 | <.001 |
| Sex: Female | 1397 (58.4%) | 370 (65.7%) | -- | <.001 |
| Education: > HS | 1020 (42.8%) | 278 (49.6%) | -- | .002 |
| BNT | 27.43 (2.02) | 21.27 (5.71) | 24.87 | <.001 |
| WFT | 36.50 (10.9) | 31.43 (11.71) | 9.20 | <.001 |
| Animal Fluency | 17.93 (4.39) | 15.15 (4.19) | 13.53 | <.001 |
| LMI | 24.02 (6.67) | 21.17 (6.43) | 9.15 | <.001 |
| LMII | 19.5 (7.12) | 15.82 (6.67) | 11.07 | <.001 |
| DWRT | 5.88 (1.44) | 5.53 (.953) | 6.66 | <.001 |
| TMT-A* | 36.89 (10.45) | 54.61 (17.26) | 22.07 | <.001 |
| TMT-B* | 92.47 (29.52) | 162.8 (60.96) | 23.99 | <.001 |
| DSST | 43.34 (9.44) | 32.6 (10.7) | 21.64 | <.001 |
| HS: High school; BNT: Boston Naming Test; WFT: Word Fluency Test; LM: Logical Memory; DWRT: Delayed word recall test; TMT: Trail Making Test; DSST: Digit symbol substitution test  Standard deviations are represented in parenthesis  * completion time in seconds | | | | |

| Supplementary Table 3: Comparison of demographic variables between LOE and Non-LOE participants stratified by race | | | |
| --- | --- | --- | --- |
|  | White Non-LOE | White LOE | Comparison |
| N | 2391 | 63 |  |
| Age | 75.47 (5.0) | 77.52 (5.11) | U= 93890, p=.002 |
| Sex: Female | 1397 (58.4%) | 36 (57.1%) | FE= .122, p=.80 |
| Education: > HS | 1020 (42.8%) | 36 (57.1%) | FE= 1.79, p=.20 |
|  | Black Non-LOE | Black LOE | Comparison |
| N | 563 | 28 |  |
| Age | 74.46 (4.82) | 78.13 (6.12) | U= 5679.5, *p*= .001 |
| Sex: Female | 370 (65.7%) | 12 (42.9%) | FE= 3.87, p=.05 |
| Education: > HS | 278 (49.6%) | 13 (46.4%) | FE= .236, p=.71 |
| LOE: late-onset epilepsy; HS: High school; FE: Fisher-Freeman-Halton exact test  Standard deviations are represented in parenthesis | | | |

| Supplementary Table 4: Comparison of cognitive scores across cognitive phenotypes | | | | | | | |
| --- | --- | --- | --- | --- | --- | --- | --- |
|  | Multidomain | Single-Domain | | Minimal |  | |  |
|  | Mean (SD) | Mean (SD) | | Mean (SD) | F | | p-value |
| **Language** | | | | | | | |
| BNT | -2.34 (2.07) | -.319 (.944) | | .046 (.88) | 22.07 | | <.001 |
| WFT | -1.33 (.771) | -.686 (.714) | | .254 (.99) | 22.49 | | <.001 |
| Animal Fluency | -1.45 (.847) | -.810 (1.01) | | .091 (1.03) | 16.59 | | <.001 |
| **Learning & Memory** | | | | | | | |
| LM1 | -1.79 (1.13) | -0.05 (.780) | | .049 (.981) | 28.45 | | <.001 |
| LM2 | -1.88 (.680) | -.185 (.717) | | .099 (1.01) | 38.47 | | <.001 |
| DWRT | -2.51(1.82) | -.892 (1.18) | | -.316 (1.2) | 16.18 | | <.001 |
| **Executive Function/Processing Speed** | | | | | | | |
| TMT-A | -4.56 (5.77) | -1.49 (1.85) | | -.026 (1.13) | 11.96 | | <.001 |
| TMT-B | -2.81 (1.99) | -2.01 (1.56) | | -.387 (1.55) | 11.02 | | <.001 |
| DSST | -1.77 (.887) | -.788 (.825) | | -.121 (.678) | 25.77 | | <.001 |
| ANOVA Pairwise group comparisons | | | | | | | |
|  | Multidomain vs Single-Domain | | Multidomain vs Minimal | | | Single-Domain vs Minimal | |
| BNT | <.001 | | <.001 | | | .92 | |
| WFT | .027 | | <.001 | | | <.001 | |
| Animal Fluency | .066 | | <.001 | | | .002 | |
| LM1 | <.001 | | <.001 | | | 1.00 | |
| LM2 | <.001 | | <.001 | | | .604 | |
| DWRT | <.001 | | <.001 | | | .369 | |
| TMT-A | .005 | | <.001 | | | .267 | |
| TMT-B | .561 | | <.001 | | | .001 | |
| DSST | <.001 | | <.001 | | | .007 | |
| BNT: Boston Naming Test; WFT: Word Fluency Test; LM: Logical Memory; DWRT: Delayed word recall test; TMT: Trail Making Test; DSST: Digit symbol substitution test | | | | | | | |
